# Comments on the genus *Diplura* C. L. Koch, 1850, with description of two new species (Araneae, Mygalomorphae, Dipluridae)

**DOI:** 10.3897/zookeys.771.24921

**Published:** 2018-07-05

**Authors:** Denis Rafael Pedroso, Alessandro Ponce De Leão Giupponi, Renner Luiz Cerqueira Baptista

**Affiliations:** 1 Museu Nacional / Universidade do Brasil (UFRJ). Quinta da Boa Vista, São Cristóvão, 20.940-040, Rio de Janeiro, RJ, Brazil; 2 Laboratório Referência Nacional em Vetores das Riquetsioses – LIRN/IOC; Coleção de Artrópodes Vetores Ápteros de Importância em Saúde das Comunidades, CAVAISC-IOC. Pav. Lauro Travassos, Anexo Posterior, sala 10. Av. Brasil, 4365, Manguinhos, Rio de Janeiro, RJ, Brazil; 3 Laboratório de Diversidade de Aracnídeos, Instituto de Biologia, Universidade do Brasil (UFRJ), Avenida Carlos Chagas Filho, 373, Ilha do Fundão, 21941-902, Rio de Janeiro, RJ, Brazil

**Keywords:** Amazonia, Atlantic Forest, biodiversity, Diplurinae, Neotropical

## Abstract

Two new species of *Diplura* C. L. Koch 1850 are described from Brazil: *Diplura mapinguari* **sp. n.**, from the state of Rondônia in southeastern Amazonia, northern Brazil, and *Diplura rodrigoi* **sp. n.**, known from southeastern and central west regions of Brazil. *Diplura rodrigoi* **sp. n.** is morphologically similar to *D. lineata* (Lucas, 1857), *D. sanguinea* (F. O. Pickard-Cambridge, 1896), and *D. mapinguari* **sp. n.** Comments on diagnostic characters of *Diplura* are included. The synonymy of *D. maculata* (Mello-Leitão, 1927) with *D. catharinensis* (Mello-Leitão, 1923) is corroborated. A classification of color pattern of the dorsum of the abdomen is given.

## Introduction

*Diplura* C. L. Koch, 1850 is a Neotropical mygalomorph genus currently including 17 species distributed from Panama to Argentina (Pedroso et al. 2016; World Spider Catalog 2018). Most species were described from southeastern and southern Brazil, with additional records from Panamá and numerous South American countries: Argentina, Bolivia, Colombia, Ecuador, Paraguay, and Venezuela (Pedroso et al. 2016). Recently, we have examined many specimens of several species from Peru. The genus is currently recognized by the following combination of characters (Pedroso et al. 2016): simple lyra, formed by a single series of strong and thickened setae, with tip spatulate and not curved (Maréchal and Marty 1998, Drolshagen and Bäckstam 2011, Pedroso and Baptista 2014), legs with thin and restricted scopula (Drolshagen and Bäckstam 2011, Pedroso and Baptista 2014), tarsi with only a few cracks (Drolshagen and Bäckstam 2011, Pedroso and Baptista 2014) and males with short and thickened palp tibia (Pedroso and Baptista 2014).

Herein we describe two new species of *Diplura*, one from the state of Rondônia, southeastern Amazonia, northern Brazil, and the other from southeastern and central west Brazil, both based on male and female specimens.

## Materials and methods

The description of color pattern is based on specimens preserved in 75% ethanol. Information and photos of living animals were included, when available. Observations, photographs and measurements were made with a Leica DFC295 camera attached to a Leica M205C stereoscopic microscope. All photos were edited in the Photoshop CS5 software and plates were prepared with CorelDraw X7 software. Measurements are given in millimeters, unless otherwise noted. Scale bars represent 1 mm, unless otherwise noted. Body length was measured from the anterior margin of the chelicerae to the posterior border of the abdomen, without spinnerets. Carapace length was measured from anterior margin of the clypeus to the posterior border. Each article of the pedipalp and legs was measured in retrolateral view, from the basal condylus to the distal one. The receptaculum seminis (spermathecae) was examined through dissection of the genital region of the females, cleaned, and immersed in clove oil for clearing. Geographical coordinates were obtained from Geonames (2018). The distribution map was created using ESRI ARCGIS 10 software.

Abbreviations. Institutions (and curators):

**CAVAISC** Coleção de Artrópodes Vetores Ápteros de Importância em Saúde das Comunidades, Instituto Oswaldo Cruz, Rio de Janeiro, Brazil (M. Amorim);

**IBSP** Instituto Butantan, São Paulo, Brazil (A. Brescovit);

**MCN** Museu de Ciências Naturais, Fundação Zoobotânica, Porto Alegre, Rio Grande do Sul, Brazil (R. Ott);

**MCTP** Museu de Ciências e Tecnologia, Pontifícia Universidade Católica, Porto Alegre, Rio Grande do Sul, Brazil (R. Teixeira);

**MNRJ** Museu Nacional, Universidade do Brasil/Universidade Federal do Rio de Janeiro, Brazil (A. Kury);

**MZSP** Museu de Zoologia da Universidade de São Paulo (R. Pinto-da-Rocha), Brazil;

**UFRJ** Laboratório de Diversidade de Aracnídeos, Instituto de Biologia, Universidade do Brasil/Universidade Federal do Rio de Janeiro, Brazil (R. Baptista);

**UNB** Universidade de Brasília, Distrito Federal, Brazil (P. Mota).

Structures:

**ALE** anterior lateral eyes;

**AME** anterior median eyes;

**ITC** inferior (unpaired) tarsal claws;

**PLE** posterior lateral eyes;

**PLS** posterior lateral spinnerets;

**PME** posterior median eyes;

**PMS** posterior median spinnerets;

**STC** superior (paired) tarsal claws.

Macrosetae:

**ap** apical;

**p** prolateral;

**pld** prolaterodorsal;

**plv** prolateroventral;

**r** retrolateral;

**rld** retrolaterodorsal;

**rlv** retrolateroventral;

**v** ventral.

## Results

### Taxonomy

#### Family DIPLURIDAE Simon, 1889

##### Genus *Diplura* C. L. Koch, 1850

###### Diplura mapinguari sp. n.

Taxon classificationAnimaliaAraneaeDipluridae

http://zoobank.org/FA9C778F-DABA-4E52-B454-8DDAB58A3FCC

Figures 1–10 , 28 , 29

####### Type material.

**Holotype**: **BraZil**: RONDÔNIA: *Porto Velho*, Parque Natural Municipal de Porto Velho, [no date], [no collector] (♂, MNRJ 04414). **Paratypes**: **BRAZIL**: RONDÔNIA: *Guajará-Mirim*, 18.i.2001, Eq. Butantan (♀, IBSP 12336); *Porto Velho*: Mutum, transecto 5, parcela 400m, 18.xi.2011, Candiani, D. (juvenile, MZSP 44043); Mutum, transecto 7, parcela 650m, 18.iv.2012, Indicatti, R. (juvenile, MZSP 47085); UHE Samuel, Reserva, 17–21.viii.1992, Pontes, G. (juvenile, MCTP 02217).

**Etymology**: The specific name is taken from the folklore of Amazonian Indian tribes. “Mapinguari” is a magical creature, a huge long-haired animal, with long arms and large claws.

####### Diagnosis.

**Figures 1–10.**
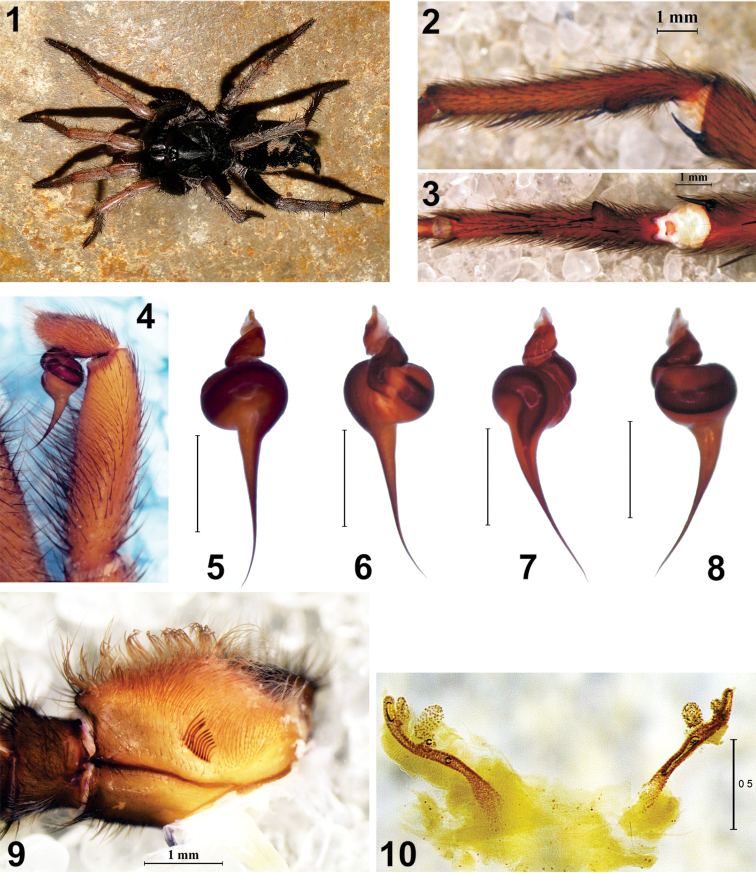
*Diplura mapinguari* sp. n. Juvenile: **1** habitus dorsal (Porto Velho-RO, photo R. Indicatti). Male: left leg I **2** tibial spur, retrolateral **3** tibial spur and metatarsal clasper, ventral **4** left palp, retrolateral; left bulb **5** retrolateral **6** prolateral **7** ventral **8** dorsal **9** maxilla with lyra. Female: **10** receptaculum seminis (dorsal).

*Diplura mapinguari* sp. n. shares the same oblique continuous light stripes on the dorsum of the abdomen with other species (*D. lineata*, *D. sanguinea*, and *D. rodrigoi* sp. n.), but differs by having the stripes unequally spaced. The three median stripes of *D. mapinguari* sp. n are wider, almost touching each other near the middle and merging with the ventral light background (Fig. 1). The male of *D. mapinguari* sp. n. has the longest embolus in *Diplura*, 2.5× longer than the bulb (Fig. 5). Females also have an elongated *receptaculum seminis*, with a thin stalk, and only three lobes: the internal lobe is lateral and larger; the other two are a pair at the apex (Fig. 10).

####### Description.

**Male (holotype, MNRJ 04414**): **Measurements**: Body length 18.2; carapace length 8.8, carapace width 7.6; abdomen length 7.5, abdomen width 3.7. Leg formula 4123, total length: I 36.2, II 31.0, III 30.2, IV 38.7. **Carapace**: Clypeus tiny, with anterior margin bearing five thick setae, elongated and turned forward. Eye tubercle without thick setae at anterior margin, but with two thick setal insertions and many thin, common setae between the posterior eyes. AME separated from each other by 1/2 their diameter and almost as large as ALE. ALE much longer than wide, just a bit longer than AME. PME oval, around 1/2 the diameter of the AME. PLE longer than wide, a bit smaller than AME. PME and PLE contiguous. Anterior and posterior eye rows slightly recurved and with similar width. Chelicera with eleven promarginal teeth. Plectrum with seven thick and elongated setae. Sigillae ellyptical. Maxillae with 17–25 digitiform cuspules. Lyra (Fig. 9) formed by eleven elongated setae, clearly curved at their middle and slightly spatulated, the basalmost seta thinner and shorter than the others, which increase regularly in length up to the distal seta. **Legs**: Leg I (Figs 2, 3). Tibia I with a retrolateral distal spur, slightly curved, with thick end which bears a megaseta. In ventral view, spur placed transversally to the long axis of tibia, with its apex directed retrolaterally (Fig. 3). Megaseta (or megaspine) elongated, regularly curved and acute, directed retrolaterally. In ventral view, it is placed obliquely in relation to its base and is nearly as long as the spur. Metatarsus I relatively long and straight, with its basal third bearing a large retrolateral tubercle, elongated and pointed, slightly turned towards the distal portion of the article. There is a short cluster of many spiniform setae (clasper) between the two macrosetae at the mid-basal portion of the prolateral face of the article. **Macrosetae**: Leg I: femur d2-3-3-3 left, d2-2-1-2-3 right; patella d1 left; tibia p1-1-1, v1-1-1ap (apophysis) left; v1-2-1ap (apophysis) right; metatarsus p1-1-0, v1-1-1-2ap left, v1-1-2-2ap right; leg II: femur d1-2-3-3-2 left, d1-1-3-3-2 right, p0-1-1-1; patella p1-1; tibia p1-1, r1, v2-2-2ap; metatarsus p1-1-1, v1-1-2-1ap left, v1-1-2-2ap right; leg III: femur d3-3-2-2; patella p1-1, r1; tibia d1-1-1, p1-1-1, r1-1-1, v2-2-2ap; metatarsus d1-1-2-2, p1-1-1, r1-1-0; v2-2-1-2ap left, v2-1-1-1-2ap right; leg IV: femur d1-3-3-1-2 left, d1-3-2-3-1-2 right; patella p1-1, r1; tibia d0-1-0, p1-1, r1-1-1, v2-1-1-2ap; metatarsus d1-1-1-1-2, p1-1-1-0, r1-1-1-0, v2-2-2-2ap left v2-2-2-3ap right. **Genitalia: Palpus** (Fig. 4) not as short and incrassate as usual, approx. 3.6× longer than wide and 2.6× longer than the cymbium. Bulb (Figs 5–8) globose, wider than long, with thin and elongated embolus, approx. 2.5× longer than the bulb. Embolus with relatively thin base, forming a 90^o^ angle with the dorsal face of bulb, in retrolateral and prolateral views, tapering regularly towards the apex, with its distal third much thinner, curved and slightly twisted. In ventral and dorsal views (Figs 7, 8), there is a clear oblique placement of the embolus axis in relation to the bulb axis. In ventral view, sperm duct is wide at the beginning, with strong diminution of diameter at the base of the embolus, and then regularly tapering toward the apex (Fig. 7).

####### Female (paratype, IBSP 12336).

**Measurements**: Body length 28.1; carapace length 11.3, carapace 9.2; abdomen length 12.3, abdomen width 8.7. Leg formula 4123, total length: I 37.3, II 34.7, III 35.2, IV 44.8. Females resemble males, except by the following characteristics. **Carapace**: Clypeus length around 1/2 the diameter of AME, with anterior margin bearing four thick setae, elongated, and turned forward. Eye tubercle with three elongated setae at anterior margin and five setae between the posterior eyes. Chelicerae with 12 promarginal teeth. Maxilae with 26 cuspules. Lyra with 13 elongated setae, slightly curved medially. Genitalia: ***Receptaculum seminis*** (Fig. 10) paired, elongated, separated by a little less than its height, with thin and elongated stalk, bearing one large and elongated internal lobe and two distal lobes that are not as large as the basal one.

####### Color pattern.

Carapace reddish brown, with thoracic sulci a bit darker. Eye region black. In live juveniles, the carapace presents an almost black color and the colors of all body parts are more vivid (Fig. 1). Chelicerae, labium, sternum and coxae reddish brown. Legs mostly reddish brown, with darker femurs. Abdomen black, dorsum with five light transverse stripes, of different sizes and shapes (Fig. 1). First and last stripes shorter and thinner (disappearing in some specimens). Three median stripes wider and shorter, widest at their middle region, where they almost touch one another. Compared to the last median stripe, the two first median stripes are wider, longer and are connected to the venter. The carapace is covered with abundant light brown setae.

####### Distribution.

Known only from Porto Velho and Guajará-Mirim, Rondônia state, Amazon area, northern Brazil (Fig. 29).

###### Diplura rodrigoi sp. n.

Taxon classificationAnimaliaAraneaeDipluridae

http://zoobank.org/8CDC2C3B-6A50-496E-B447-4820C19F50AF

Figures 11–20 , 25 , 29

####### Type-material.

**Holotype: BRAZIL**: RIO DE JANEIRO: *Casimiro de Abreu*: BR-101, xii.2010, Equipe Herpetologia(♂, MNRJ 7620, ex. UFRJ 0920). **Paratypes: BRAZIL**: RIO DE JANEIRO: *Campos dos Goytacazes*: Mata do Mergulhão, 10.iii-05.iv.2004, Teixeira, C. L. (8♂, MCN/FZBRS 43436); Mata do Mergulhão, 10.iii-05.iv.2004, Teixeira, C. L. (8♂, MCN/FZBRS 43437); *Casimiro de Abreu*: BR-101, xii.2010, Equipe Herpetologia (♂, UFRJ 0920); Barra de São João, Morro de São João, 21–24.iii.2003, Exp. Arachné (♂, MNRJ 4344); *Macaé*: Terminal Cabiúnas, 25–30.iii.2010, Baptista, R. et al. (♂, MNRJ 4539); 21–26.ii.2013, Pedroso, D. R. and Miranda, G. (♂, UFRJ MAC 3617); 19–24.ii.2016, Pedroso, D. R. and Villarreal, O. (♂, CAVAISC, ex. UFRJ); 19–24.ii.2016, Pedroso, D. R. and Villarreal, O. (♂, CAVAISC, ex. UFRJ); *Mendes*: Colégio Marista São João das Paineiras, v.2008, Baptista, R. (♀, UFRJ 0102); *Rio de Janeiro*: Parque Nacional da Tijuca, Bom Retiro, v.2016, Pedroso, D. R. and Baptista, R. L. C. (♂, UFRJ 1360); vi.2016, Pedroso, D. R. and Baptista, R. L. C. (♂, MNRJ 7620, ex. UFRJ 1361).

####### Etymology.

This species is named after the biologist, Rodrigo de Cerqueira da Costa, who first directed Denis Pedroso and Alessandro Giupponi in their studies of zoology.

####### Diagnosis.

*Diplura rodrigoi* sp. n. differs from other species displaying several oblique continuous light stripes on the dorsum of the abdomen (*D. lineata*, *D. sanguinea*, and *Diplura mapinguari* sp. n.) by its very characteristic stripe pattern. Its elongated oblique stripes are very thin near the median line of the dorsum but continuously widening and approaching each other towards the venter, where they merge in the light background (Fig. 25). The three median stripes are longer and not as wide and confluent at their middle portion as in *Diplura mapinguari* sp. n. In contrast to *D. lineata* and *D. sanguinea*, the light stripes are longer and clearly delimited, with well-defined borders. Males of *D. rodrigoi* sp. n. are the only Diplurinae with a prolateroventral swelling at the distal portion of metatarsus I, which is glabrous and lighter than the surrounding areas (Fig. 13). As in *D. sanguinea*, males of *Diplura rodrigoi* sp. n. bear a relatively wide sperm duct from the base of the embolus towards the apex (Fig. 17), in contrast to *Diplura mapinguari* sp. n. and *D. lineata*, where the sperm duct is strongly constricted and becomes almost filiform towards the apex. *Diplura rodrigoi* sp. n. males differ from *D. sanguinea* by having the megaseta of tibial spur of leg I a bit shorter than the basal portion of the spur (Fig. 12) and 10–11 setae in the lyra (Fig. 19), in contrast with megaseta longer than the spur and seven setae at the lyra in the latter species.

**Figures 11–20.**
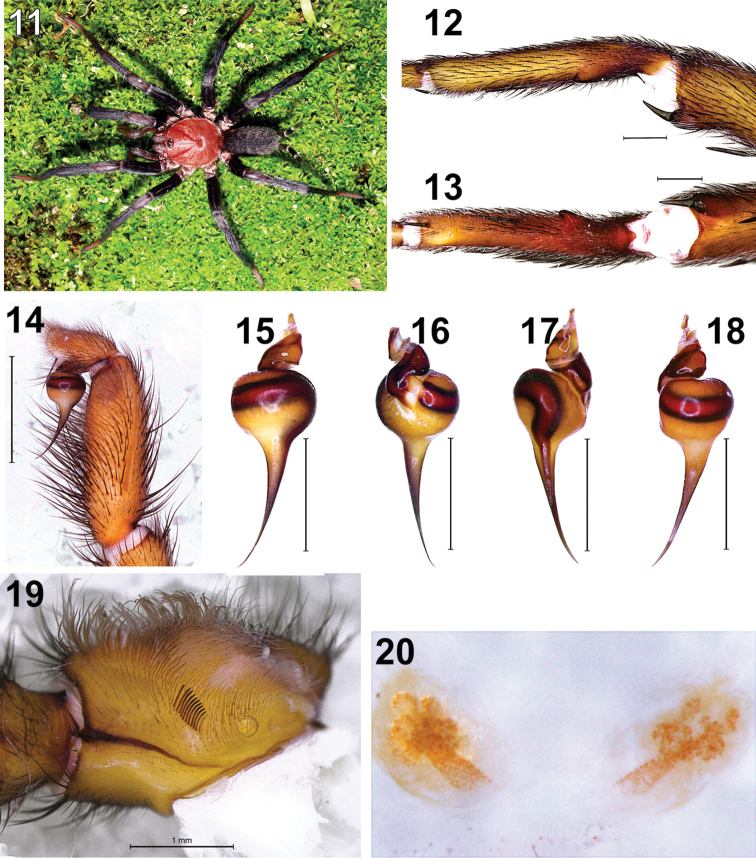
*Diplura rodrigoi* sp. n. Juvenile: **11** habitus dorsal (Porto Velho-RO, photo R. Indicatti). Male: left leg I **12** tibial spur, retrolateral **13** tibial spur and metatarsal clasper, ventral **14** left palp, retrolateral; left bulb **15** retrolateral **16** prolateral **17** ventral **18** dorsal **19** maxilla with lyra. Female: **20** receptaculum seminis (dorsal).

####### Description.

**Male** (Holotype, **MNRJ 7620)**: **Measurements**: Body length 20.0; carapace length 8.7, width 7.2. Abdomen length 8.3, width 4.8. Leg formula 4123, length: I 33.3, II 30.6, III 27.4, IV 35.0. **Carapace**: Clypeus about 40% the diameter of AME, partially covered by eye tubercle; frontal margin bearing five thick setae, which are elongated and turned forward. Eye tubercle with four thick setae at anterior margin and two setae between posterior eyes. AME separated from each other by 1/3 their diameter, and a little larger than ALE. ALE longer than wide. PME almost spherical, with around 1/2 the AME diameter. PLE longer than wide, a little shorter than AME diameter. PME and PLE contiguous. Anterior eye row a little recurved, posterior row recurved. Anterior and posterior rows of similar width. Chelicera with 12 promarginal teeth. Plectrum with six thick and elongated setae. Sigillae ellyptical. Maxillae with 12–15 elongated cuspules. Lyra (Fig. 19) formed by eleven setae almost straight or just a bit curved, of similar size, with apex slightly spatulate, the basalmost seta much thinner than the others. **Legs**: Leg I (Figs 12–13). Tibia I with distal retrolateral spur slightly curved, not acutely pointed, bearing an apical megaseta, much longer than wide. Spur megaseta short and slightly curved, a little shorter than the spur. Metatarsus I relatively long and slightly sinuous in ventral view, bearing a retrolateral tubercle conical, pointed and turned in apical direction, placed at the basal third of the article. Cluster of spiniform setae (clasper) placed from the base to the level of the third megaseta at the mid-basal region of the prolateral face of the article. Swelling placed near the prolateral distal margin, glabrous and lighter than the surrounding areas. **Megasetae**: leg I: femur d1-2-1-3-3 left, d1-1-1-1-2 right; patella p1 left; tibia p1-1, v1-1-1ap (apophysis); metatarsus p0-1-1 left, p1-1-1 right, v0-1-1ap; leg II: femur d2-3-2-2-1 left, d2-3-3-2 right; patella p1; tibia p1-1, v1-2-2ap; metatarsus p1-1-1, v1-2-2ap; leg III: femur d2-3-2-2 left, d1-3-2-3-2 right; patella p1 left, p1-1 right, r1; tibia d1-1, p1-1, r1-1, v2-1-1-2ap; metatarsus d1-1-2, p1-1-1, r1-1-1-1 left, r1-1-1-0 right; v1-1-1-1-3ap; leg IV: femur d1-1-2-3-2-2 left, d1-1-2-2-3-2 right; patella p1 left, p1-1 right, r1; tibia d1-1, p1-1, r1-1, v2-1-1-2ap; metatarsus d1-1-0-2 left, d1-1-1-2 right, p1-1-0 left, p1-1-1 right, r1-1-1, v1-1-1-2-3ap left v2-1-1-2-3ap right. Genitalia: Palpus (Fig. 14) short and incrassate, around 3× longer than wide and 2.6× longer than the cymbium. Bulb (Figs 15–18) almost piriform, a little wider than long, with embolus moderately long and thin, around 2× longer than bulb. Embolus with base relatively thick in relation to the bulb. In ventral view, sperm duct wide at the beginning, with a moderate diminution of diameter at the base of the embolus, and then tapering toward the apex, with distal third much thinner (Fig. 17). Embolus regularly and slightly curved, especially at the distal region.

**Female** (Paratype, UFRJ 0102): **Measurements**: Body length 26.4; carapace length 9.6, carapace width 8.2; abdomen length 12.3; abdomen width 7.6. Leg formula 4123, length: I 30.4, II 24.8, III 23.7, IV 30.6. Females resemble males, except by the following characteristics. **Carapace**: Clypeus length similar to AME diameter, with anterior margin bearing five thick setae, elongated and turned forward. Eye tubercle with three thick and long setae and ten smaller setae, of variable size, at anterior margin. Area between posterior eyes with single long and thick setae. AME separated from each other by 80% of their diameter. Chelicera with 12–13 promarginal teeth. Plectrum with five thick and elongated setae. Maxillae with 15–17 cuspules. Lyra (Fig. 19) formed by 11–12 subequal setae, except for the first one, which is shorter and thinner. Setae slightly spatulated, with a thin and pointed apex. **Genitalia**: *Receptaculum seminis* (Fig. 20) paired, separated by a distance similar to its own height. Stalk relatively wide, its diameter a little smaller at the distal third, near the five or six apical large lobes, with all of them being similar in size.

####### Color pattern.

Carapace reddish, with thoracic sulci a little darker. Eye region darkened. Chelicerae reddish brown. Labium, sternum and coxae orange brown. Sigillae slightly darker than sternum. Legs light brown. Dorsum of abdomen dark brown with five elongated pale brown transverse stripes, with their width diminishing posteriorly. Stripes continuous, with well-defined borders, thin and far from each other near the midline of the dorsum, but growing wide and closer toward the venter, where they blend into the pale background. When alive, they bear a vivid red carapace and dark brown legs and abdomen.

####### Natural history.

The female paratype from Mendes (UFRJ 0102) was collected in a short tunnel, with a small silk lined opening, on the slope of an old road through second-growth Atlantic Forest. Other specimens have been collected under rocks and logs on forest floor. Most specimens were caught by pitfall traps in forested areas.

####### Records.

**BRAZIL**: DISTRITO FEDERAL: *Brasília*: Reserva Ecológica do IBGE/RECOR: alt. 1077m, -15.939653° -47.879984°, 14–16.v.2015, Kury, A., Pinto, A. and Carvalho, R. (♂, MNRJ 6859); IBGE, mata de galeria, 23.iv.2001, Diniz, D. (2♂, UNB 1142); 01.v.2000, Milhomem, M. (2♂, UNB 3998); ESPÍRITO SANTO: *Santa Teresa*: ESFA leg. (♂, MNRJ 4339); *Sooretama*: Reserva Biológica de Sooretama, Porteira Quirinão, 20.iv.2006, Exp. Arachné (♂, MNRJ 4341); GOIÁS: *Morrinhos*: Parque Ecológico Jatobá, XII.2006-VIII.2007, Santana, R. (♂, IBSP 140891); MINAS GERAIS: *Belo Horizonte*, Estação Ecológica da UFMG, iii.2001, Maria, M. et al. (♂, IBSP 10739); UFMG, campus Pampulha, 2000, Álvares, E. (♂, IBSP 13824); *Itacarambi*: Gruta Olhos d´água, 26.vi.2001, Giupponi, A. and Baptista, R. (♂, MNRJ 3518); *Juiz de Fora*: ix.2008-iv.2009, Gomide, S. (♂, ♀, IBSP 144024; ♂, IBSP 144025); *Viçosa*: Mata do Paraíso, 21–24.iii.2001, Azevedo, L. P. (♂, MNRJ 4320); RIO DE JANEIRO: *Macaé*: Ilha de Santana, 27.vi.2009, Baptista, R. L. C. (j, UFRJ MAC 2166; j, UFRJ MAC 2246); Parque Municipal do Atalaia, 26.vi.2009, Baptista, R. L. C. et al. (2 j, UFRJ MAC 2174); Terminal Cabiúnas, Mata da Odebei, 11–16.v.2015, Pedroso, D. R. and Castanheira, P., pitfall (♂, UFRJ MAC 10265); *idem*, 18–22.ii.2014, Pedroso, D. R. and Castanheira, P., pitfall (♂, UFRJ MAC 7191); SÃO PAULO: *Peruíbe*: Estação Ecologica Juréia-Itatins, III.1997, Brescovit, A. et al. (♂, IBSP 11577).

**Distribution**: Widespread and common in a wide area of southeastern and mid-western Brazil, from north Espírito Santo state and central-south Minas Gerais state, reaching west to the Distrito Federal and south, down to southeastern São Paulo state (Fig. 29). It is sympatric with *D. lineata* (Lucas, 1957), a similar species, in the municipalities of Casimiro de Abreu and Rio de Janeiro, both in Rio de Janeiro state, Brazil (see Pedroso et al. 2016).

## Discussion

### Notes on the diagnosis and composition of *Diplura*

The diagnosis of *Diplura* in relation to other Diplurinae needs some comments and amendments. The presence of a lyra in the inner face of the palp maxilla is a key character that allows for separation between lyrate (*Diplura* C. L. Koch, 1850, *Harmonicon* F. O. Pickard-Cambridge, 1896 and *Trechona* C. L. Koch, 1850) and alyrate Diplurinae (*Linothele* Karsch, 1879). The single line of stiff setae found in lyra of *Diplura* and *Harmonicon* is quite different from the plate-like lyra, with several series of setae, in *Trechona* (Pedroso et al., 2008). In *Harmonicon*, the lyra is formed by four to seven setae in adult specimens, with a flattened and curved tip (Drolshagen and Bäckstam 2011, Pedroso and Baptista 2014). In *Diplura*, the number of setae in the lyra is quite variable. The described species of *Diplura* have stiff setae in the lyra ranging from four to seven in *Diplura taunayi* (Mello-Leitão, 1923) to 15 in *Diplura studiosa* (Mello-Leitão, 1920). However, we found specimens of *Diplura* from southern Brazil with up to 18 setae. On the other hand, specimens from southeastern and northeastern Brazil may have only one to three stiff setae in the lyra. One specimen we examined had only one seta in one maxilla, but the other maxilla was devoid of stiff setae. Those findings show that the number of setae in the lyra may increase or decrease in different species, and even the complete absence of the lyra may happen in some specimens. Therefore, as pointed out by Drolshagen and Bäckstam (2011) and Pedroso and Baptista (2014), the number of stiff setae in the lyra is not diagnostic for the genera.

The shape of the stiff setae of the lyra is much more variable than previously pointed out in the literature. In many described species of *Harmonicon*, including the type-species *Harmonicon rufescens* F. P.-Cambridge, 1896, the setae in the lyra are strongly hook-shaped, with a strong distal curvature and an acute and not widened tip (F. P.-Cambridge, 1896: pl. 35, fig. 3, Maréchal and Marty 1998: fig. 2, Pedroso and Baptista 2014: fig. 6). However, the lyra of *Harmonicon oiapoqueae* Drolshagen & Bäckstam, 2011 does not display a clear hook, with the tip less curved and a little widened (Drolshagen and Bäckstam 2011: fig. 6), similar to some undescribed species we have examined from Brazil. The lyra of one of those undescribed species has a very wide and clearly spatulated tip. On the other hand, there are species of *Diplura* with curved stiff setae (ex. *D. mapinguari* sp. n., Fig. 9) and the tip not clearly spatulated (see *D. lineata*, Pedroso et al. 2016: fig. 22–24, and *D. rodrigoi* sp. n., Fig. 19). Therefore, we consider that the variation found on the shape of the stiff setae of the lyra in both *Diplura* and *Harmonicon* are too wide and clearly overlapping.

Besides the lyra, there are also other diagnostic characters in the maxilla. On its ventral face, there is a transversal suture just below the lyra. In *Diplura*, the area just below the basal portion of the maxillar suture is either glabrous or has just some small thin setae (Figs 9, 19). On the other hand, there is a field of spiniform setae in that area in *Harmonicon* (Pedroso and Baptista 2014: fig. 6) and *Trechona* (Guadanucci et al. 2016: fig. 1g). Additionally, there is also a dense fringe of long and thin setae between the lyra and the basal portion of the suture in *Trechona* (Guadanucci et al. 2016: fig. 1g).

Specimens of *Diplura* have relatively short and thin scopula, sometimes almost absent in the metatarsus and tarsus of the legs. Scopulae in the other lyrate Diplurinae are formed by long numerous setae and cover most of the distal articles of the legs. In *Diplura*, the tarsi of the legs are not pseudosegmented and not very flexible, lacking large and numerous cracks; they have only a few ventral, thin cracks, that allow just a limited bending of the article (Drolshagen and Bäckstam 2011, Pedroso and Baptista 2014).

**Figures 21–22.**
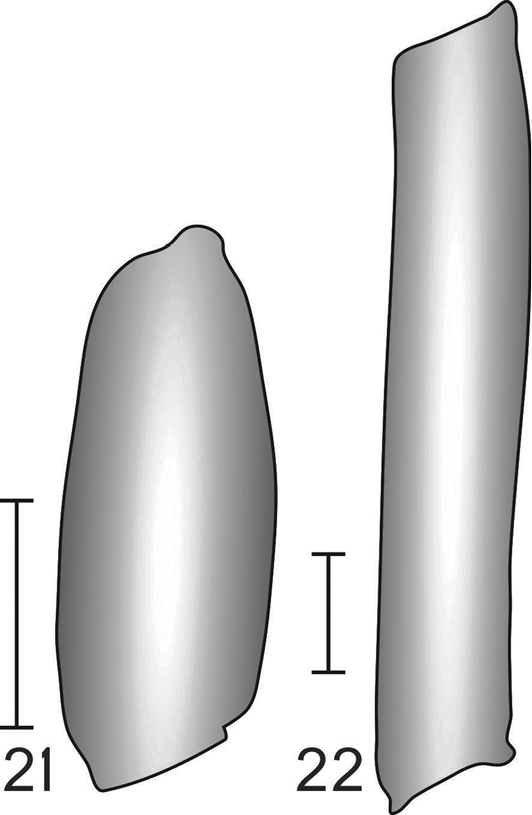
Pedipalp tibia drawings of male Diplurinae (what view): **21** *Diplura* sp. **22** *Harmonicon* sp.

An easy way to separate males of *Diplura* from other Diplurinae is by the short and incrassate pedipalp tibia (Fig. 21), which contrasts strongly with the elongated and thin tibia of other Diplurinae (Fig. 22). The ratio length/width of the pedipalp tibia reaches 3.6× in *Diplura* and is at least 5× in the other Diplurinae. This character was already pointed out by Pedroso and Baptista (2014) and Pedroso et al. (2016).

The *receptaculum seminis* of most *Diplura* species is formed by a short and relatively wide stalk topped by a cluster of many globular to ovoid lobes (*fundus*), of variable sizes (Fig. 20, Pedroso et al. 2016: fig. 13, 25). In *D. mapinguari* sp. n., the stalk is elongated and thinner and the lobes are separated in two groups (Fig. 10). In *Harmonicon* (Drolshagen and Bäckstam 2011: fig. 5) and *Trechona* (Pedroso and Baptista 2004: fig. 1, Pedroso et al. 2008: fig. 4), there are usually two branches: the first one simple and at the inner side of the stalk, and the second one distal and topped by one large fundus, sometimes with lateral flaps. However, there are no clear multiple lobes as in *Diplura*.

Currently, 17 species of *Diplura* are considered as valid, distributed from Panama to Argentina (Pedroso et al. 2016; World Spider, Catalog 2018). However, *Diplura maculata* (Mello-Leitão, 1937) has been already synonymized with *Diplura catharinensis* (Mello-Leitão, 1927) by Bücherl (1962: 261). Although this synonymy had been completely overlooked in the literature and catalogs, we agree with Bücherl after examining the types and additional material from the type-localities or nearby areas in Santa Catarina state, southern Brazil. As a result, *Diplura* now includes 18 species, taking into consideration the synonymy referred to above and the two new Brazilian species herein described.

**Figures 23–28.**
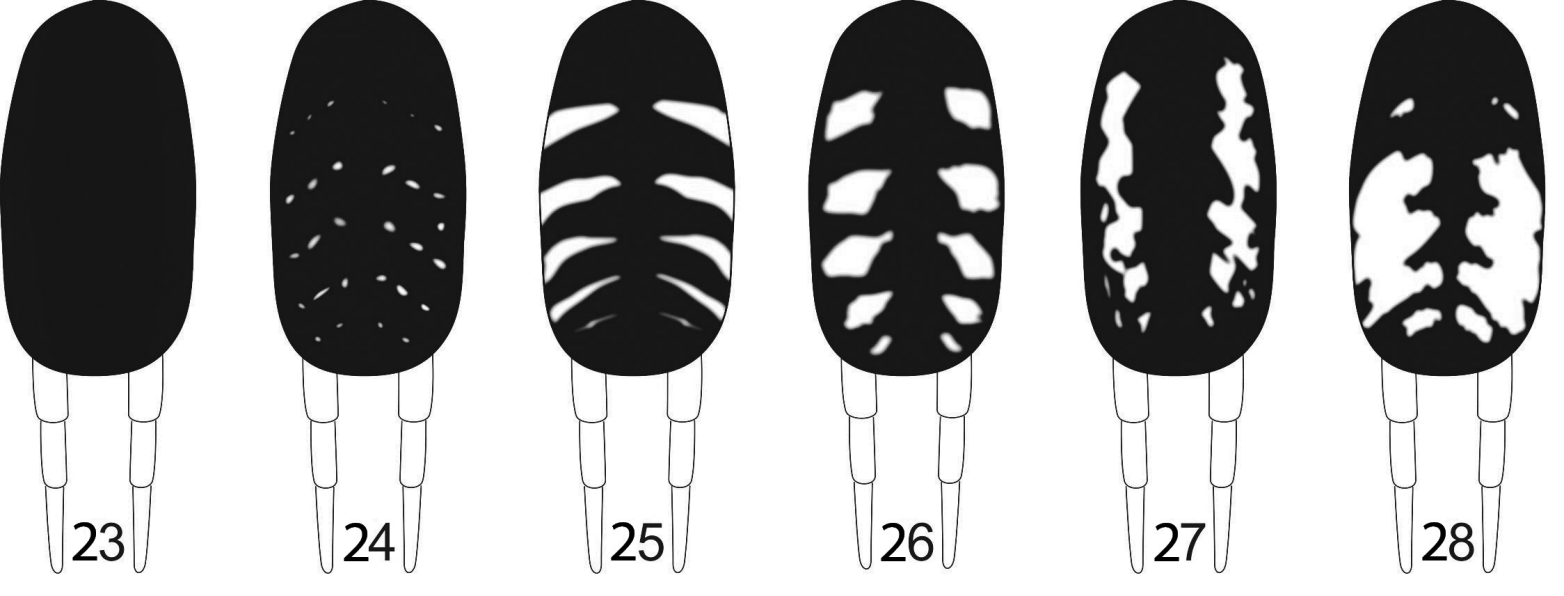
Drawings of color pattern types of the dorsum of the abdomen in *Diplura* species: **23** Type I **24** Type II **25** Type III **26** Type IV **27** Type V **28** Type VI.

Most Diplurinae species have a pattern of light stripes or dots over the dark dorsum of the abdomen. The exceptions are *Harmonicon*, and some species of *Diplura*, whose abdomen are uniformly dark, without a contrasting light pattern. The light pattern of most Diplurinae may be present only at the sides of the dorsum, but usually extend either to the middle area of the dorsum or to the venter, even merging with the background color in that area. The species of *Diplura* may be grouped below in six color pattern types following the different markings on the dorsum of abdomen, numbered from I to VI (Figs 23–28). These pattern types are an easy way to group species and also allows a clear distinction between these groups or isolated species.

**Figure 29.**
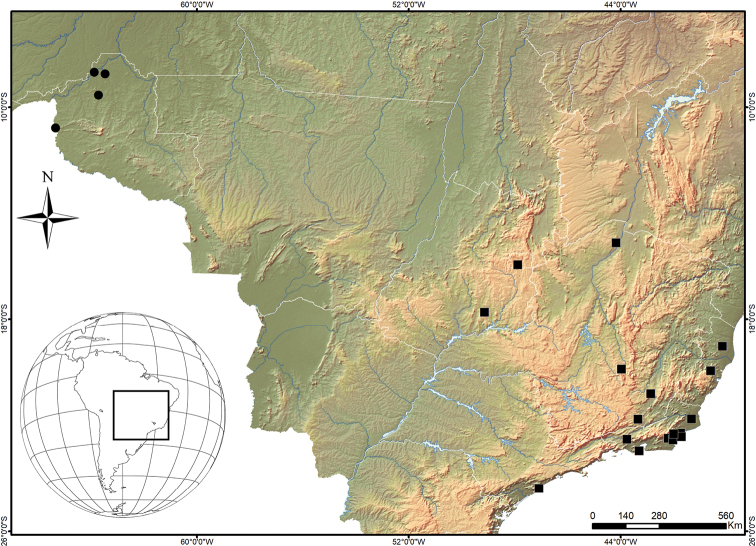
Geographical distribution of the two new species of *Diplura*. Key: black circles *Diplura mapinguari* sp. n., black squares *Diplura rodrigoi* sp. n.

**Type I** (Fig. 23): Uniform dark color, sometimes with sparse and inconspicuous light dots randomly spread: *D. annectens* (Bertkau, 1880), *D. macrura* (C. L. Koch, 1841), *D. paraguayensis* (Gerschman & Schiapelli, 1940), *D. riveti* (Simon, 1903), *D. studiosa* (Mello-Leitão, 1923).

**Type II** (Fig. 24): Pattern with many light dots randomly distributed on the dorsum or with a variable number (2–6) of “pseudostripes”, where many dots are aligned but not fused: *D. argentina* (Canals, 1931), *D. catharinensis* (Mello-Leitão, 1927), *D. erlandi* (Tullgren, 1905), *D. nigra* (F. O. Pickard-Cambridge, 1896), *D. paralella* (Mello-Leitão, 1923), *D. taunayi* (Mello-Leitão, 1923).

**Type III** (Fig. 25): Pattern formed by continuous and well-defined transversal stripes, with thin tips near the middle area of dorsum, but increasing in width towards the venter, each stripe with a more or less triangular profile: *D. rodrigoi* sp. n.

**Type IV** (Fig. 26): Pattern formed by elongated transverse light blotches, sometimes more or less restricted to the sides of the dorsum: *D. garbei* (Mello-Leitão, 1923), *D. petrunkevitchi* (Caporiacco, 1955), *D. sanguinea* (F. O. Pickard-Cambridge, 1896).

**Type V** (Fig. 27): Pattern formed by irregular large light spots, with most of them connected to one another, forming an irregular longitudinal stripe on the sides of the dorsum: *D. lineata* (Lucas, 1857).

**Type VI** (Fig. 28): Pattern formed by three irregular very wide light spots, widest at their middle region, where they almost touch one another: *D. mapinguari* sp. n.

The only described species of *Diplura* not listed above is *D. garleppi* (Simon, 1892), from San Mateo, Bolivia. As its holotype has not been found in the Museum national d´Histoire naturelle, Paris (E. Leguin, pers. comm.), the short description does not give enough detail on the color pattern and we have not examined any specimen ascribable to *D. garleppi*, we were not able to include it in any of the proposed color pattern types.

## Supplementary Material

XML Treatment for Diplura mapinguari

XML Treatment for Diplura rodrigoi
